# Endodontic–sinus communications: a 10-year retrospective observational study on maxillary posterior teeth

**DOI:** 10.1007/s00784-026-06958-w

**Published:** 2026-06-05

**Authors:** Calogero Bugea, Francesca Cerutti, Francesco Sforza, Enrico Nicola Sciancalepore, Stanislav Heranin, Eugenio Pedullà, Erda Qorri, Antonio Scarano

**Affiliations:** 1 Private Practice, Gallipoli, LE Italy; 2 Private Practice, Lovere, BG Italy; 3 Private Practice, Carovigno, BR Italy; 4 Private Practice, Molfetta, BA Italy; 5 Department of Dentistry, School of Medicine II, V. N. Karazin Kharkiv National University, Kharkiv, Ukraine; 6 Department of General Surgery and Surgical-Medical Specialties, University of Catania, Catania, Italy; 7 Department of Dentistry, Faculty of Medical Sciences, Albanian University, Tirana, 1001 Albania; 8 Department of Medical, Oral and Biotechnological Sciences, University of Chieti-Pescara, Chieti, Italy; 9 Lungomare G. Galilei 133B, Gallipoli, LE 73014 Italy

**Keywords:** Endodontic–sinus communication, Maxillary sinus, Posterior maxillary teeth, Iatrogenic complications, Sodium hypochlorite accident, Bioceramic sealer, Endodontic treatment

## Abstract

**Objectives:**

To describe the clinical characteristics, anatomical distribution, and long-term outcomes of unintended endodontic–sinus communication involving maxillary posterior teeth that occurred during endodontic treatment.

**Materials and methods:**

This retrospective observational study reviewed clinical records collected over approximately 10 years of routine clinical practice. Among 10,345 maxillary premolars and molars treated endodontically, cases presenting intraoperative endodontic–sinus communication were identified. Preoperative periapical radiographs were obtained in all cases, with cone-beam computed tomography (CBCT) available in selected patients. Endodontic treatment was performed under rubber dam isolation using electronic apex locators and rotary nickel–titanium instruments. The Valsalva maneuver was systematically performed intraoperatively to detect communication. Root canal obturation was completed in one or two visits using bioceramic techniques. Clinical and radiographic follow-up was scheduled at 3, 5, and 10 years.

**Results:**

Thirty-one patients (18 males, 13 females) presented an endodontic–sinus communication, corresponding to an incidence of 0.30% (31/10,345). The affected teeth included 14 maxillary second molars, 15 maxillary first molars, and 2 maxillary second premolars. A total of 32 root-level communications were identified. Vital primary treatments accounted for 22 cases (70.97%), retreatments for 7 cases (22.58%), and necrotic teeth for 2 cases (6.45%). The Valsalva maneuver was positive in all cases. The mean follow-up duration was 48 months (range: 36–120 months). All evaluated teeth remained functional, with no persistent sinus-related symptoms and no need for surgical intervention.

**Conclusions:**

Unintended endodontic–sinus communication is a rare intraoperative event during treatment of maxillary posterior teeth. Early recognition, strict apical control, and conservative management may allow favorable long-term outcomes without the need for surgical intervention. However, these findings should be interpreted cautiously in light of the retrospective design, limited sample size, and absence of a control group.

## Introduction

CBCT studies have consistently demonstrated a close anatomical relationship between the roots of maxillary posterior teeth and the maxillary sinus floor. A substantial proportion of maxillary premolars and molars present root apices in direct contact with, or protruding into, the sinus cavity, with molar teeth—particularly palatal roots—being most frequently involved [1–5]. This anatomical relationship may also be appreciated on conventional periapical radiographs. Clinically significant oroantral communications have been described in association with posterior maxillary teeth, highlighting the relevance of this anatomical configuration [6]. Beyond endodontics, the maxillary sinus has been extensively investigated in implantology, with particular attention to its anatomy, biological behavior, and surgical complications related to sinus floor elevation procedures [7–11]. In endodontic literature, proximity between posterior maxillary root apices and the sinus has traditionally been discussed mainly in relation to procedural complications, such as overfilling or extrusion of irrigants, with potential sinus involvement [11, 12]. Consequently, strict respect of apical limits during instrumentation has been emphasized. Nevertheless, unintended endodontic–sinus communication may still occur during canal shaping, even when preventive measures are adopted [13]. Sudden changes in electrical impedance near an air-filled cavity may affect working length determination and increase the risk of over-instrumentation. Available evidence addressing this complication remains limited and is largely confined to isolated case reports describing sodium hypochlorite penetration into the maxillary sinus and its acute clinical manifestations [14–17]. Therefore, the aim of the present retrospective observational study was to describe the clinical characteristics, anatomical distribution, and long-term outcomes of unintended endodontic–sinus communication occurring during endodontic treatment of maxillary posterior teeth, and to propose a pragmatic intraoperative decision-making approach for its conservative management.

## Materials and methods

### Study design

This retrospective observational study reviewed clinical records of patients treated during routine clinical practice over an observation period of approximately 10 years. The large denominator of treated maxillary posterior teeth (10,345 cases) allowed a reliable estimation of the observed incidence of endodontic–sinus communication despite the limited number of events. Endodontic treatments were performed in two private endodontic practices: one located in Gallipoli, Apulia, Italy, and one located in Lovere, Lombardy, Italy. All endodontic procedures included in the study were carried out by two experienced operators with specific expertise in endodontics, C.B. (Gallipoli practice) and F.C. (Lovere practice), following standardized clinical and diagnostic protocols that were routinely adopted throughout the observation period.

### Case selection and preoperative assessment

**Fig. 1:**
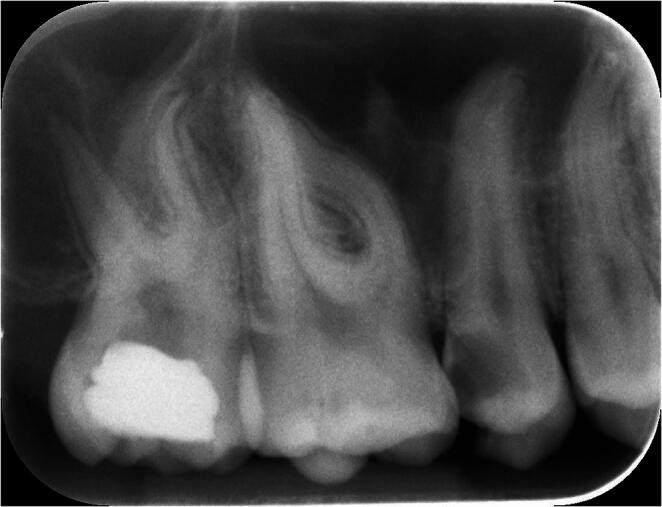
Preoperative periapical radiograph Preoperative periapical radiograph showing close anatomical proximity between the apices of maxillary posterior teeth and the maxillary sinus floor.

All included teeth required endodontic treatment, including primary treatments and retreatments of both vital and necrotic teeth. During the study period, a total of 10,345 maxillary premolars and molars underwent endodontic treatment in the two participating clinical settings. A preoperative periapical radiograph was obtained in all cases before treatment. In some patients, radiographic findings suggested a close anatomical relationship or possible communication between the root apex and the maxillary sinus, whereas in other cases no evident radiographic signs were observed (Fig.1). CBCT examinations were available only in selected patients and had been previously prescribed for unrelated diagnostic or surgical indications, mainly implant planning or third molar assessment. No CBCT examination was prescribed specifically to investigate the relationship between the tooth apex and the maxillary sinus. However, when available, CBCT images were reviewed by the operators after conventional periapical radiographic evaluation in order to obtain a more detailed understanding of the local anatomy before treatment. During routine treatment of maxillary posterior teeth, a standardized intraoperative diagnostic protocol was systematically adopted throughout the observation period. Following canal shaping, the Valsalva maneuver was routinely performed in all cases to assess the possible presence of endodontic–sinus communication. In addition, the pulp chamber and root canal system were carefully inspected under magnification for indirect signs suggestive of sinus communication, including excessive bleeding, sudden disappearance of irrigant from the pulp chamber, or synchronized blood movement associated with respiratory cycles. Cases were included when an unintended endodontic–sinus communication was identified intraoperatively on the basis of a positive Valsalva maneuver associated with direct clinical evidence of air, blood, mucous, or irrigant passage through the root canal system. Both primary treatments and retreatment cases were included. Cases with incomplete clinical records, uncertain diagnosis, pre-existing oroantral communication, previous sinus surgery, or lack of follow-up data were excluded from the analysis.

### Endodontic treatment protocol

All procedures were performed under rubber dam isolation. Working length determination was carried out using electronic apex locators (Dental Port ZX or Tri Auto ZX2; J. Morita Corp., Kyoto, Japan). Root canal shaping was performed using ProTaper Gold (Dentsply Maillefer, Ballaigues, Switzerland) nickel–titanium instruments. At the end of canal shaping, the Valsalva maneuver was systematically performed in all cases as part of a standardized intraoperative diagnostic protocol to assess the possible presence of endodontic–sinus communication. The maneuver was performed by asking the patient to gently exhale against closed nostrils while maintaining oral opening, allowing the operator to detect possible air or fluid passage through the root canal system. The diagnostic procedure was performed according to the same clinical protocol in all cases [15]. Root canal obturation was performed in one or two visits using either a single-cone technique with bioceramic sealer or a coneless bioceramic-based obturation technique. Ceraseal (Meta Biomed, South Korea) was used as the root canal sealer. No intracanal medicament was used. When a second visit was required, temporary sealing was achieved using polytetrafluoroethylene tape and IRM (Dentsply Maillefer, Ballaigues, Switzerland). The overall clinical and diagnostic protocol remained substantially unchanged throughout the entire observation period. Irrigation was performed using sodium hypochlorite with careful apical control in order to minimize the risk of irrigant extrusion toward the maxillary sinus. When an endodontic–sinus communication was detected, irrigation procedures were carried out using apical negative-pressure irrigation systems (EndoVac; Kerr Endodontics, Orange, CA, USA, or iVac; NSK-Nakanishi Inc., Tochigi, Japan) in order to reduce the risk of irrigant extrusion. Root canal obturation was performed according to the bioceramic obturation protocols previously described by the authors [18]. A postoperative periapical radiograph was obtained at the end of treatment.

### Follow-up

Patients with a positive Valsalva maneuver were enrolled in a dedicated follow-up protocol with clinical and radiographic evaluations scheduled at 3, 5, and 10 years. Clinical success was defined as the absence of pain, swelling, sinus-related symptoms, tenderness to percussion, or need for further surgical intervention. Radiographic success was defined as the absence of progressive periapical radiolucency or other radiographic signs suggestive of persistent disease during follow-up evaluations.

### Statistical analysis

Descriptive statistics were used to summarize demographic characteristics, anatomical distribution, pulpal status, intraoperative findings, and follow-up outcomes. Continuous variables were reported as mean values and ranges, whereas categorical variables were expressed as absolute frequencies and percentages. Given the retrospective design and limited sample size, no inferential statistical tests were performed. All analyses were carried out using Microsoft Excel (Microsoft Corporation, Redmond, WA, USA).

### Ethical statement

This retrospective observational study was conducted in accordance with the Declaration of Helsinki and the General Data Protection Regulation (EU 2016/679). Ethical approval was obtained from the Albanian University Ethics Committee (Protocol No. 275, September 24, 2018). All clinical data were anonymized prior to analysis. Written informed consent for treatment and anonymous use of clinical data for scientific purposes was routinely obtained from all patients. Clinical records were reviewed retrospectively by the investigators, and all patient identifiers were removed before data extraction and analysis in order to ensure confidentiality and data protection.

### Results

Among the 10,345 treated maxillary premolars and molars, 31 patients presented an unintended endodontic–sinus communication. This corresponded to an incidence of 0.30% (31/10,345 treated maxillary premolars and molars). The study population consisted of 18 males and 13 females.

### Pulpal status

Of the 31 teeth, 22 were vital (70.97%), 7 were retreatment cases (22.58%), and 2 were necrotic (6.45%). Among the second premolars, one was vital and one was a retreatment case.

### Anatomical distribution

The affected teeth included 14 maxillary second molars (45.2%), 15 maxillary first molars (48.4%), and 2 maxillary second premolars (6.4%). A total of 32 root-level communications were identified.

- Second premolars: palatal root involvement in both cases (100%).
- First molars: palatal root in 9 cases (56.3%), mesiobuccal root in 5 cases (31.3%), distobuccal root in 2 cases (12.4%); one tooth showed dual palatal and distobuccal involvement.
- Second molars: mesiobuccal root in 6 cases (42.9%), palatal root in 5 cases (35.7%), distobuccal root in 3 cases (21.4%).

Both maxillary molars and premolars were included in the present series, although communications were more frequently observed in molars. No substantial differences in the clinical management approach were adopted according to tooth type.

### Intraoperative findings

The Valsalva maneuver was positive in all cases. Only air passage was observed in 15 cases (48.4%), blood passage in 12 cases (38.7%), mucous discharge in 3 cases (9.7%), and purulent discharge in 1 case (3.2%). In 12 cases, blood flow from the canal disappeared synchronously with respiration. In 5 cases, irrigant passage toward the sinus with nasal outflow was reported.

### Follow-up outcomes

Eight cases were completed in a single visit; the remaining cases required two visits. One patient was lost during the follow-up period and one patient died from unrelated causes. All remaining patients completed the minimum scheduled follow-up evaluation of 36 months. Four patients reached a 120-month follow-up, while the remaining cases underwent intermediate follow-up evaluations according to the treatment period. The mean follow-up duration for the evaluated cases was 48 months (range: 36–120 months). All evaluated teeth remained functional and asymptomatic, with no persistent sinus-related symptoms and no need for surgical intervention.

### Discussion

The present retrospective observational study suggests that unintended endodontic–sinus communication may occur during endodontic treatment of maxillary posterior teeth. Although relatively uncommon, its clinical relevance lies in the potential complications that may arise if communication is not promptly recognized and managed. Existing literature addressing this condition is limited and mainly confined to isolated case reports, often focusing on sodium hypochlorite-related accidents [12–14, 19, 20]. These reports describe acute sinonasal symptoms following irrigant penetration into the maxillary sinus but provide limited guidance regarding intraoperative recognition, prevention, or conservative management strategies. Anatomical proximity between posterior maxillary root apices and the sinus floor represents the principal predisposing factor [1–6, 20]. CBCT-based studies have consistently shown that maxillary molars, particularly palatal roots, are most frequently involved, a finding that is in agreement with the anatomical distribution observed in the present series. Both maxillary molars and premolars were included in the present series, although communications were more frequently observed in molars. No substantial differences in the clinical management approach were adopted according to tooth type.

The Valsalva maneuver proved to be a simple, effective tool for intraoperative detection of sinus communication. The presence of air passage or fluid movement during the maneuver allowed immediate recognition and adoption of a conservative management strategy. Performing the maneuver during canal shaping enables early identification of communication, before extensive irrigation or obturation procedures are undertaken. The present findings suggest that endodontic–sinus communication may become clinically evident at different stages of endodontic treatment and with variable intraoperative manifestations, ranging from isolated air passage to blood, mucous, or irrigant extrusion. Early intraoperative identification appears particularly important, as recognition of communication during the initial phases of canal shaping may allow immediate modification of irrigation and instrumentation strategies before accidental sodium hypochlorite extrusion toward the maxillary sinus occurs. In this regard, the Valsalva maneuver may represent a simple and clinically useful method for early detection before more significant sinonasal complications develop.

Failure to recognize communication early may result in intraoperative complications, including sudden bleeding from the root canal system with disappearance synchronized with respiration. This phenomenon suggests the presence of negative pressure within the sinus cavity, which may facilitate accidental aspiration of irrigants toward the sinus. Penetration of sodium hypochlorite into the maxillary sinus can result in direct chemical injury to the Schneiderian membrane and acute sinonasal symptoms [12–14, 19, 20]. Patients may report unusual taste sensations, coughing, or sneezing, and irrigant exiting from the nose is highly distressing and may significantly undermine confidence in the dental treatment. Moreover, postoperative complications such as epistaxis or rhinorrhea may occur even when the intraoperative event is not immediately recognized [20].

In one case in the present series, the endodontic–sinus communication was not identified during the early stages of treatment, and accidental overextension of the instrumentation toward the maxillary sinus was clinically suspected. The patient developed significant epistaxis a few hours after treatment, associated with severe pain that persisted for several days. Conservative management with tranexamic acid and external cooling measures was recommended. The bleeding resolved within a few hours, while pain progressively decreased and was markedly reduced after approximately 2 days. Although no specific intervention was required, this episode further emphasized the importance of early intraoperative recognition of communication in order to prevent more severe sinonasal complications.

When canal dryness could not be achieved due to persistent bleeding or mucous exudate, definitive obturation was postponed. Accurate documentation of working length was essential, as variations may occur between appointments, particularly in the presence of periapical inflammation [21]. Selection of the master gutta-percha cone during the first visit facilitated safe and controlled obturation at the subsequent appointment. In cases of suspected irrigant extrusion, irrigation was continued using negative-pressure systems, avoiding positive-pressure delivery, in order to minimize further extrusion of irrigants toward the sinus cavity [20, 22, 23]. This approach may reduce the risk of exacerbating chemical injury and associated symptoms.

**Fig. 2:**
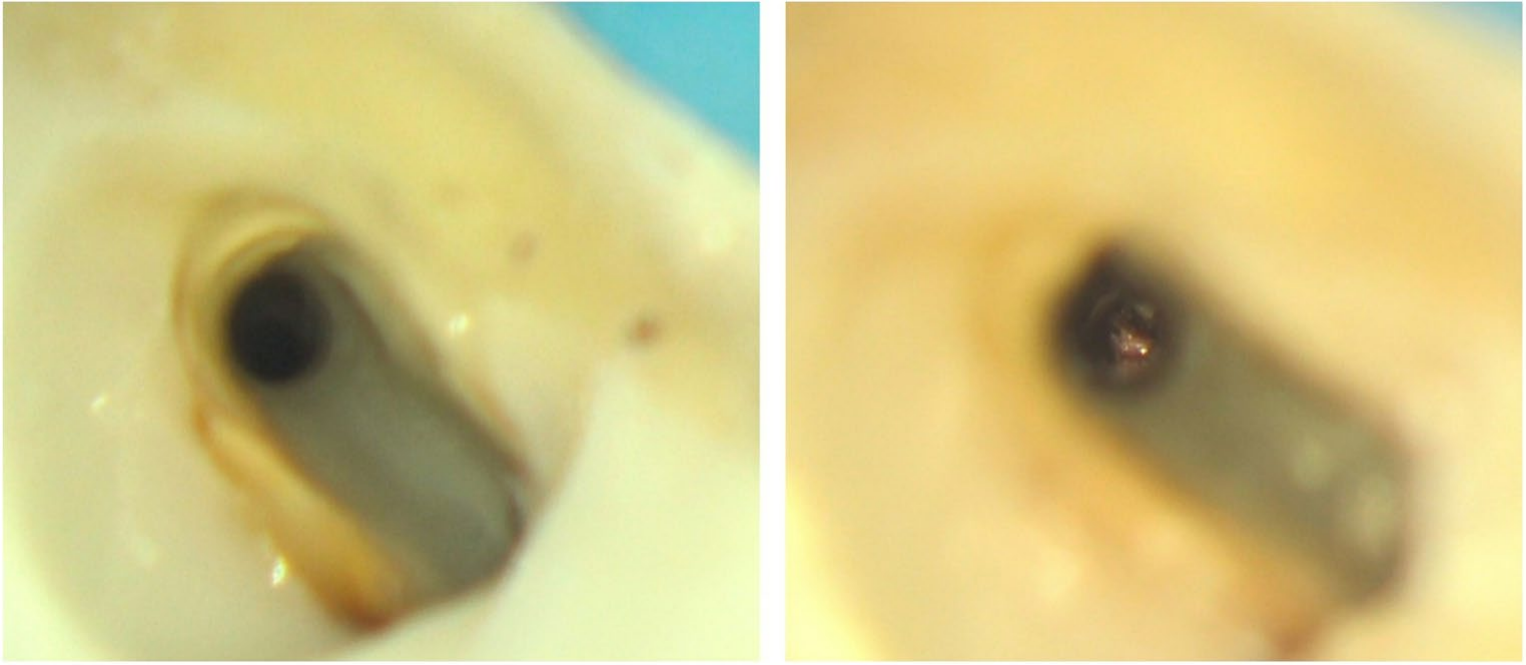
Microscopic images of an endodontic–sinus communication at the apical level. The left image shows the communication immediately after intraoperative detection, with direct visualization of the sinus cavity. The right image shows the same site one week later, suggesting an early tissue response at the communication site. No histologic confirmation was available regarding the nature of the observed tissue changes

Microscopic observation suggested an early tissue response at the site of communication within a short time interval, supporting a conservative, staged approach to management. However, no histologic confirmation was available regarding the nature of the observed tissue changes. This finding is consistent with previous observations of sinus membrane healing capacity reported in both implant and regenerative surgery literature [24, 25] (Fig. 2).

When CBCT imaging was available, it provided additional anatomical information regarding the relationship between the root apex and the maxillary sinus. In the present series, 13 patients had a pre-existing CBCT examination available for review. However, only a limited number of cases showed CBCT findings that appeared to influence the clinical management or modify the planned endodontic treatment strategy. No clear indication emerged for routinely prescribing CBCT solely on the basis of conventional radiographic signs suggestive of sinus communication, especially when a careful intraoperative diagnostic protocol is systematically adopted.

**Fig. 3:**
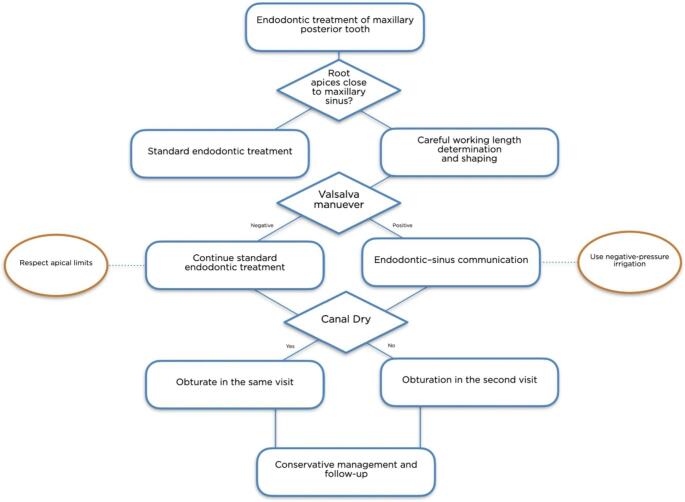
Proposed intraoperative decision-making pathway for the recognition and conservative management of endodontic–sinus communication during endodontic treatment of maxillary posterior teeth. The figure summarizes the clinical steps recommended following working length determination, including the use of the Valsalva maneuver, interpretation of intraoperative findings, and subsequent management strategies aimed at minimizing complications and preventing irrigant extrusion into the maxillary sinus

Finally, odontogenic involvement in maxillary sinus disease has been widely recognized, and dental-origin sinus complications may be misinterpreted or overtreated when the dental cause is not promptly identified [26, 27]. The absence of structured clinical guidance for managing intraoperative endodontic–sinus communication highlights the need for practical decision-making tools. On the basis of the present clinical experience, a pragmatic intraoperative decision-making pathway is proposed, aimed at minimizing complications and preserving patient trust while avoiding unnecessary surgical interventions [13] (Fig. 3).

The present study has several limitations that should be acknowledged. First, its retrospective observational design may introduce selection and reporting biases. Second, the relatively small number of cases reflects the rarity of the condition and limits the possibility of performing statistical comparisons or identifying prognostic factors. In addition, the absence of a control group and the non-systematic availability of CBCT imaging may have influenced the interpretation of some anatomical findings. Intraoperative identification of endodontic–sinus communication partially relied on operator-dependent clinical observations, although a standardized diagnostic protocol was consistently adopted throughout the study period. Although no formal inter-operator calibration analysis was performed due to the retrospective nature of the study, all procedures and intraoperative assessments were conducted by two experienced operators following the same standardized diagnostic and clinical protocol throughout the observation period. Although the overall clinical and diagnostic protocol remained substantially consistent throughout the observation period, minor unrecognized variability in clinical procedures over time cannot be completely excluded. In addition, the reported incidence may have been influenced by possible under-detection of minor communications, and some degree of operator-dependent variability in intraoperative detection cannot be completely excluded despite the use of a standardized diagnostic protocol. Despite these limitations, the present series represents one of the largest clinical observations currently available regarding unintended endodontic–sinus communication and provides clinically relevant information regarding its recognition and conservative management.

## Conclusions

Endodontic–sinus communication is a rare but clinically relevant intraoperative event during treatment of maxillary posterior teeth. Early recognition, strict apical control, and conservative management a may allow favorable long-term outcomes without the need for surgical intervention. However, these findings should be interpreted cautiously in light of the retrospective design, limited sample size, and absence of a control group.

## Data Availability

The data supporting the findings of this study are available from the corresponding author upon reasonable request.
